# Supplementary material for: Differences in Troponin I and Troponin T Release in High-Performance Athletes Outside of Competition
Source: Int J Mol Sci. 2024 Jan 15;25(2):1062. doi: 10.3390/ijms25021062 (PMC10816948; doi:10.3390/ijms25021062)
Supplement: Supplementary file 1 [file ijms-25-01062-s001.zip › ijms-2788217-supplementary.pdf]

International Journal of
Molecular Sciences

MDPI

Supplemental Data:

Table S1. Individuals per sporting discipline (all/women/men).

Biathlon (51/24/27)	*Running (<800m) (4/3/1)*	*Wrestling (8/1/7)*
Bobsledge (12/3/9)	*Running (800-1500m) (8/1/7)*	*Ludge (1/0/1)*
Boxing (1/0/1)	*Running (>1500m) (7/3/4)*	*Rowing (1/1/0)*
Curling (1/1/0)	*Long-jump (1/1/0)*	*Shooting (2/0/2)*
Judo (1/1/0)	*Nordic combined (1/0/1)*	*Swimming (5/1/4)*
Canoe (race) (9/5/4)	*Para-throw (1/0/1)*	*XC Skiing (49/24/25)*
Karate (1/1/0)	*Para-game sport (1/1/0)*	*Ski Jumping (7/7/0)*
Discus throw (4/1/3)	*Para-triathlon (1/0/1)*	*Speedskating (2/0/2)*
Race walk (1/1/0)	*Track cycling (2/0/2)*	*Triathlon (12/4/8)*
Hammer throw (7/5/2)	*Mountainbike (1/0/1)*	*Water diving (4/2/2)*
Shotput (1/0/1)	*Road cycling (2/0/2)*	*Fencing (1/0/1)*
Fin swimming (2/0/2)	*Handball (4/1/3)*	*Track and Field multicomp. (1/1/0)*
